# Sharing Unpleasant Health Information with Patients: A baseline study exploring physician attitudes, practices and adherence to the SPIKES protocol at a tertiary hospital in Muscat, Oman

**DOI:** 10.18295/squmj.10.2023.063

**Published:** 2024-08-29

**Authors:** Rahma Al Kindi, Hajar Al Mamari, Asma Al Salmani, Rahma Al Hadhrami, Adhari Al Zaabi

**Affiliations:** 1 Department of Family Medicine & Public Health, Sultan Qaboos University Hospital, University Medical City, Muscat, Oman; 2 College of Medicine and Health Sciences, Sultan Qaboos University, Muscat, Oman; 3 Department of Human & Clinical Anatomy, Sultan Qaboos University, Muscat, Oman

**Keywords:** Physician-Patient Relations, Truth Disclosure, Clinical Protocols, Communication, Empathy, Oman

## Abstract

**Objectives:**

This study aimed to investigate the knowledge, attitude and experiences in sharing unpleasant health information and adherence to the SPIKES protocol among physicians at a tertiary hospital in Muscat, Oman.

**Methods:**

This cross-sectional study was conducted at the Sultan Qaboos University Hospital from August to October 2022. An electronic, self-administered questionnaire was used to gather data from physicians across various departments.

**Results:**

A total of 89 physicians completed the questionnaire (response rate = 22.3%). Most participants (n = 86, 96.6%) recognised the need for additional training in the delivery of unpleasant health information (‘bad news’), with 78.7% (n = 70) expressing their willingness to undertake such training. Additionally, 32.6% (n = 29) reported negative experiences due to improper delivery of bad news, with an equal proportion admitting to disclosing bad news to patients’ family without their consent. The majority (n = 77, 86.5%) demonstrated a high level of overall adherence to the SPIKES protocol, with 59.6–85.4%, 12.4–34.8% and 1.1–11.2% reported usually, sometimes and never following specific steps of the protocol, respectively. Marital status (*P* = 0.015) and qualifications (*P* = 0.032) were the only variables that were associated with adherence level, with married physicians and those with board and/or fellowship certificates reporting significantly better adherence compared to their counterparts.

**Conclusion:**

Physicians in Oman encounter challenges in delivering unpleasant health information, underscoring the interplay of cultural influences, training and adherence to protocols. To address these challenges, targeted and frequent training programmes are recommended, starting from undergraduate medical education and extending to continuous opportunities for physicians at various career levels.

**Advances in Knowledge**

*- This study found that, although most physicians reported prior experience in conveying unpleasant health information and receiving education and training in this area, the majority indicated the necessity for additional training to enhance their skills. Moreover, one-third of the participants disclosed negative experiences due to the improper delivery of such news, with a similar proportion admitting to having first disclosed confidential information to a patient’s family without the patient’s consent*.
*- To the best of the authors’ knowledge, this study is the first in Oman to evaluate physician knowledge, experiences and attitudes regarding the delivery of unpleasant health updates to patients*.

**Applications to Patient Care**

*- This study’s findings provide useful information which could inform future educational campaigns and initiatives to improve the delivery of unpleasant health information to patients by physicians. This can potentially enhance physician-patient communication and trust, improving patients’ satisfaction with their care and fostering adherence to treatment and follow-up*.
*- The authors strongly advocate for the integration of comprehensive communication skills training into undergraduate medical education and postgraduate residency training, as well as the provision of regular refresher courses to ensure that physicians across all medical specialties can deliver unpleasant health information to patients with appropriate sensitivity, accuracy and empathy*.

Physicians must acquire a broad range of skills during their studies and training, with communication skills ranking among the most crucial.^1^ This skill-set becomes especially vital as physicians navigate numerous challenges throughout their careers, including the demanding task of delivering unpleasant health information (‘bad news’) to patients. In the context of healthcare information, bad news refers to “any news that the doctor announces to the patient which [could] have the ability to shock the patient and destroy his [or her] hopes, resulting in changing his lifestyle and thoughts about his future”.^2^ This encompasses informing a patient or their relatives of the development, recurrence or spread of various life-altering or even life-threatening diagnoses, such as cancer, degenerative neurological conditions, advanced heart disease, infertility and HIV infection/AIDS.^2^ Other situations may involve conveying unfavourable information regarding a patient’s prognosis, treatment failure, test results and adverse complications or side-effects and engaging in end-of-life discussions.

Delivering unpleasant health information is a crucial aspect of patient-provider communication and significantly influences patients’ satisfaction with their care, shaping their perception of their illness and their compliance with medical treatment.^3^^,^^4^ Indeed, research has shown a direct correlation between physicians’ communication skills and therapeutic outcomes.^4^^,^^5^ Improperly delivered bad news can result in negative consequences for patients, families and physicians alike, adversely impacting patients’ level of trust in their healthcare providers.^5^ Research has shown that patients often prioritise perceived physician empathy over their clinical performance.^6^^,^^7^ Additionally, some physicians, particularly those with less experience, have expressed a need for additional training in delivering unpleasant health information, possibly due to their fear of the patient’s emotional reaction and of evoking blame or due to their lack of experience in conveying distressing information with compassion.^8^^,^^9^

It is therefore crucial that healthcare professionals approach these conversations with honesty, compassion and sensitivity, employing clear and concise language while offering appropriate support and resources to help patients cope with the emotional and practical challenges associated with such news. Additionally, involving patients in the decision-making process is crucial in fostering shared decision-making and patient-centred care. Several protocols, developed by experts, aim to guide physicians in delivering unpleasant health information effectively.^10^–^12^ Notably, the SPIKES protocol, widely adopted in clinical practice, comprises 6 key steps: (1) setting (choosing a private, comfortable location for the conversation); (2) perception (assessing the patient’s readiness to receive the news and existing awareness of their condition or situation); (3) invitation (asking the patient how much information they desire or seeking clarification of any doubts); (4) knowledge (providing key information about the diagnosis and treatment options in clear, concise and simple language); (5) emotion (addressing and accepting the patient’s reaction with empathy and providing emotional support); and (6) strategy (delivering the diagnosis, outlining the treatment plan or any next steps and arranging a follow-up appointment).^11^

The delivery of unpleasant health information to patients by physicians in the Middle Eastern region is under-researched; to the best of the authors’ knowledge, no studies have been conducted in Oman regarding physicians’ utilisation of the SPIKES protocol. It remains unclear whether physicians in Oman adhere to the SPIKES protocol or if they employ alternative approaches with similar objectives. Additionally, physicians’ adherence to such protocols may be influenced by various sociocultural factors, such as their medical training, cultural background and the customs and traditions of the patient population they serve. As such, this study aimed to explore the knowledge, attitude and experiences of physicians working at a tertiary hospital in Muscat, Oman, in relation to the delivery of unpleasant health information and assess their level of adherence to the SPIKES protocol.

## Methods

This cross-sectional study was conducted at the Sultan Qaboos University Hospital (SQUH) from August to October 2022. Employing a total population sampling strategy, the study targeted all physicians (including medical officers, specialists, senior specialists, consultants and senior consultants) practicing in patient-facing specialities at SQUH, such as the medicine, paediatric, urology, oncology, surgery, nephrology and orthopaedic specialties. Physicians in fields without direct patient contact, such as radiologists and histopathologists, were excluded from the study.

Data were obtained from the participants using an electronic, self-administered questionnaire published online using Google Forms (Google LLC, Mountain View, California, USA). A link to the online questionnaire was disseminated via email to doctors across the various departments at SQUH. The questionnaire comprised 4 main sections. The first section focused on gathering information regarding the participants’ sociodemographic characteristics, including their age, gender, marital status, qualifications, clinical position, medical specialty and number of years of work experience.

The second section featured a previously reported 9-item English-language questionnaire related to the participating physicians’ level of knowledge, training and experience in the delivery of unpleasant health updates.^13^ The questions included topics such as previous training in breaking bad news, perceived need for training in skill development, willingness to attend future training, prior experience in breaking bad news to patients or their families, instances of negative experiences from improper delivery of bad news, preference for communicating directly with patients or their family members when breaking bad news, belief regarding the direct delivery of bad news to affected patients, instances of breaking bad news to patients’ families without patients’ consent and instances of delivering bad news to patients by telephone rather than in person. Additionally, a concise definition of ‘bad news’ was provided.

The third section consisted of 6 items designed to assess the physicians’ adherence to the SPIKES protocol for breaking bad news.^11^^,^^13^ Responses to each item were scored on a 3-point Likert scale based on frequency of adherence to each step of the protocol (usually, sometimes or never). Total scores ranged from 0–12, with a score of 12 indicating perfect adherence.^13^ For the purpose of the current study, total scores of <6, 6–8 and ≥9 indicated low, medium and high levels of adherence to the SPIKES protocol.

The fourth and final section of the questionnaire consisted of 25 items designed to explore each respondent’s opinions regarding the delivery of unpleasant health information. Responses were scored on a 5-point Likert scale based on the physicians’ level of agreement with each statement (strongly disagree, disagree, not sure, agree or strongly agree).

The Statistical Package for the Social Sciences (SPSS) software, Version 27.0 (IBM Corp. Armonk, New York, USA), was used for all statistical analyses. Sociodemographic characteristics were reported using descriptive statistics. For categorical variables, frequencies and percentages were reported, while for continuous variables, means and standard deviations were used. Associations between independent and outcome variables were estimated using an independent samples t-test and Chi-squared test. A *P* value of <0.05 was considered statistically significant.

Ethical approval for this study was obtained in July 2022 from the Medical Research and Ethics Committee of the College of Medicine and Health Sciences, Sultan Qaboos University, Muscat, Oman (REF. NO. SQU-EC/146/2022). Prior to completing the questionnaire, written informed consent was obtained from all participants. Participants were provided with detailed information about the study’s main aim and objectives and were informed that participation was entirely voluntary. At the commencement of the questionnaire, all participant rights were clearly stated, including the right to withdraw at any time. Participating physicians were assured that the survey did not intend to provide medical advice and that all collected information would be treated with strict confidentiality. All responses were coded and stored in a secure database accessible only to the researchers.

## Results

A total of 89 physicians working in patient-facing specialties at SQUH completed the questionnaire and were included in this study (response rate = 22.3%). Among the respondents, 45 (50.6%) were male and 44 (49.4%) were female. The mean age was 38.0 ± 10.0 years (range: 22–60 years), with most participants (n = 54; 60.7%) being ≤40 years old. Regarding clinical position, participants were most frequently house officers (n = 32, 36.0%), followed by specialists (n = 22, 24.7%), senior consultants (n = 18, 20.2%), senior specialists (n = 10, 11.2%) and consultants (n = 7, 7.9%). The most commonly represented specialty was internal medicine (n = 37, 41.6%), followed by surgery (n = 14, 15.7%), paediatrics (n = 12, 13.5%), behavioural medicine (n = 10, 11.2%), family medicine (n = 9, 10.1%) and obstetrics and gynaecology (n = 7, 7.9%). The mean number of years of work experience was 12.5 ± 9.4 years (range: 1–30 years) [Table 1].

The majority of participants (n = 77, 86.5%) reported having had prior experience in breaking bad news to patients, with a considerable proportion (n = 72, 80.9%) indicating that they had received education and training in this regard. The vast majority agreed that training was necessary for physicians to develop adequate skills in breaking bad news (n = 86, 96.6%) and expressed a willingness to attend future training for this purpose (n = 70, 78.7%). Almost one-third of the participants (n = 29, 32.6%) reported having had negative experiences with patients as a result of improperly delivering bad news. Similarly, an equal proportion (n = 29, 32.6%) admitted to first disclosing unpleasant health information to a patient’s family without the patient’s consent, and the majority (n = 73, 82.0%) agreed that such news should be delivered directly to the patient. A small proportion of respondents (n = 9, 10.1%) admitted to delivering bad news to patients via telephone rather than in-person [Table 2].

Usual adherence to each step in the SPIKES protocol was reported by 59.6–85.4% of the respondents; however, 12.4–34.8% and 1.1–11.2% reported sometimes and never adhering to specific steps of the protocol, respectively [Table 3]. The mean adherence score was 10.28 ± 2.07 (range: 0–12, median score = 11). A perfect score was reported by 29 (32.6%) doctors [Table 4]. Overall, low, medium and high adherence to the SPIKES protocol was reported by 2 (2.2%), 10 (11.2%) and 77 (86.5%) participants, respectively [Table 5]. Significant correlations were observed between level of adherence to the SPIKES protocol and the respondents’ marital status (*P* = 0.015) and qualifications (*P* = 0.032). Specifically, married physicians and those with board and/or fellowship certificates reported significantly higher adherence scores compared to their respective counterparts. No significant associations with any other sociodemographic or clinical characteristics were found [Table 6].

## Discussion

Breaking bad news is a crucial communication skill for doctors working in medical fields with regular patient contact.^14^^,^^15^ A recent meta-analysis of qualitative studies focusing on healthcare practitioners’ experiences with delivering such news highlighted the emotionally distressing nature of this task, often causing discomfort and relational distress.^16^ Other studies have indicated that delivering bad news can elicit a physiological stress response, along with emotions of anxiety, self-blame, fatigue, a sense of failure and frustration.^17^^,^^18^ A global survey of healthcare practitioners working in hospitals across 40 countries and 5 continents revealed that only 33.4% had received formal training in delivering bad news to patients.^19^ Unfortunately, younger practitioners and those with fewer years of work experience were more likely to be involved in delivering bad news to patients, despite being statistically less likely to have received formal training in this area.^19^

In the current study, most of the surveyed physicians (80.9%) admitted to having received prior training in delivering unpleasant health information to patients. These findings are in line with research conducted in Egypt and Brazil, likely reflecting the increased integration of relevant training in this regard into medical school curricula.^20^^,^^21^ However, it is noteworthy that medical schools often prioritise imparting medical knowledge over training students in the development of practical communication skills. While the responses from participants in the present study indicate an awareness of the general guidelines regarding the delivery of unpleasant health information, a proportion of the respondents were unaware that their usual methods of delivering bad news to patients required adherence to a specific protocol.

Incidents of improperly delivering bad news are not uncommon among physicians. In the current study, 32.6% of the surveyed doctors at SQUH reported negative experiences as a result of this, mirroring findings from studies conducted in Sudan, Korea and Nigeria.^13^^,^^22^^,^^23^ This issue often stems from a lack of training and awareness. Communication skills related to giving bad news have historically been overlooked in global medical school curricula. Only recently has the importance of teaching these skills as an essential component of a doctor’s education been recognised.^24^ Nonetheless, it is important to acknowledge that education alone is insufficient, and accompanying training is essential.^25^ Proper training in the delivery of bad news not only reduces the anxiety associated with this task but also enhances physician self-confidence and self-efficacy.^26^–^28^ In the current study, an overwhelming majority of the respondents (96.6%) agreed that training is necessary for developing adequate skills in delivering bad news. This aligns with the results of a study conducted in Sudan, in which 94.8% of the participating doctors expressed a similar sentiment.^13^

Social and cultural influences play a significant role in the delivery of health information to patients, often outweighing professional considerations.^29^^,^^30^ Notably, substantial differences exist between Eastern and Western cultures concerning family involvement in medical decision-making.^31^ In Western societies, individualism emphasises the importance of personal autonomy, while the collectivist cultures in the East prioritise familial relationships and group harmony.^32^ Furthermore, cultural and religious beliefs strongly influence healthcare preferences in the East, with family members contributing to decisions based on their shared values and traditions.^33^ In Oman, previous studies have affirmed considerable family involvement in healthcare decision-making, even as far as withholding the diagnosis itself from the patient.^34^^,^^35^

This dynamic might explain why 18.0% of the participants in the current study believed that unpleasant health information should be disclosed to relatives directly, with 32.6% of them admitting to have disclosed confidential information directly to a patient’s family without the patient’s consent. A study conducted in Saudi Arabia, a neighbouring country to Oman, similarly found that 70% of physicians preferred to discuss information with close relatives rather than patients; moreover, in cases of serious disease, 32% admitted that they would inform the patient’s family without the patient’s consent.^29^ Comparatively, studies from Sudan and Egypt have reported higher percentages of participants who preferred to share bad news with the patients’ family (34.4% and 59.2%, respectively).^13^^,^^20^ In contrast, 82.0% of participants in the current study acknowledged the importance of maintaining the patient’s rights to confidentiality and autonomy, advocating for the direct delivery of bad news to patients.

In Omani culture, family cohesion is highly valued, leading some doctors to disclose bad news directly to patients’ families and sometimes overlook the patient’s individual rights as defined in Royal Decree 75/2019, a law which outlines the guidelines for practice in various medical professions.^36^^,^^37^ Specifically, Article 12 of this decree stipulates that a medical practitioner must disclose to the patient the nature and seriousness of their illness.^37^ However, if this is not in the patient’s best interest—for instance, in cases where the patient is incapacitated or too unwell to fully comprehend their health situation—the information may be conveyed to a second-degree relative. Emphasising adherence to medical law is pivotal in upholding patients’ rights to safety, autonomy and confidentiality as well as in protecting healthcare practitioners from liability. Notably, in cases concerning child health, the responsibility often falls upon healthcare providers to convey distressing information directly to the family as the child is considered a minor under law and therefore legally incapable of making their own healthcare decisions.

Overall adherence to the SPIKES protocol in the current study was high, with 59.6–85.4% of the respondents reporting that they usually follow each of the 6 steps of the SPIKES protocol. However, different studies have indicated variables rates of adherence to individual steps of the protocol. For example, a study of Sudanese doctors showed that 35–79% were usually adherent to each step of the SPIKES protocol.^13^ Another study involving Korean doctors indicated that 80% believed they correctly followed the SPIKES protocol when delivering difficult news to their patients.^22^ The current study revealed no significant correlations between adherence to the SPIKES protocol and most of the participants’ demographic or clinical characteristics, including gender, age and number of years of work experience. These findings align with the results of studies conducted in Sudan, Egypt and Saudi Arabia, which also did not observe significant correlations between adherence to the SPIKES protocol and these factors.^13^^,^^20^^,^^29^ However, marital status and qualifications were both found to significantly influence level of adherence in the present cohort.

It is possible that married physicians might possess enhanced communication skills, empathy and emotional intelligence because of their experience in maintaining effective interpersonal relationships; this skill-set could translate into better communication with patients and their families.^38^^,^^39^ Moreover, married physicians may draw from their own personal experiences and emotions related to family dynamics, making them more attuned to others’ emotional needs. In turn, the process of pursuing advanced qualifications might equip physicians with the necessary tools to navigate sensitive conversations, including additional training in communication skills development or prior experience with the SPIKES protocol itself. However, further research is necessary to corroborate these findings and determine how and why such factors might influence adherence to the SPIKES protocol among physicians in Oman.

A major strength of the current study is its distinction as the first in Oman to assess physicians’ practices and adherence to the SPIKES protocol in the delivery of unpleasant health information to patients. However, several important limitations should be acknowledged. First, the low response rate could have introduced sampling bias. Second, the self-administrated nature of the questionnaire could have potentially impacted the results due to social desirability and memory recall biases among the respondents. Third, the cross-sectional study design prevents the establishment of temporality. Fourth, the SPIKES protocol is only intended to guide doctors on the important steps to take when delivering bad news to patients; therefore, rigid adherence to the protocol is not always warranted in every clinical situation. Finally, this research was conducted at a single hospital setting in Oman, limiting the generalisability of the results to the entire population. Future multi-centre studies with a larger sample size involving doctors from various hospitals and health centres in Oman are recommended.

## Conclusion

While majority of the surveyed physicians had received prior training in breaking bad news, a considerable proportion reported negative experiences resulting from the improper delivery of such news. Similarly, a notable number admitted to disclosing health information to their patient’s family without the patient’s consent. These findings highlight the complex interplay between cultural influences, training and adherence to protocol in the delivery of unpleasant health information by physicians in Oman. To address these challenges, the authors recommend frequent, targeted training to equip healthcare practitioners with the essential knowledge and skills to effectively and empathetically communicate bad news to patients. Such training should be integrated into undergraduate medical curricula from an early stage. Furthermore, providing opportunities for refresher training to physicians across diverse medical specialties and at all career levels is essential as it will foster continuous improvement in this critical aspect of physician-patient communication.

## Figures and Tables

**Table 1:** Characteristic of physicians included in this study (N = 89)

Characteristic	n (%)
**Gender**	
Male	45 (50.6)
Female	44 (49.4)
**Age in years**	
≤40	54 (60.7)
>40	35 (39.3)
Mean ± SD (range)	38.0 ± 10.0 (22–60)
**Marital status**	
Single	21 (23.6)
Ever been married	68 (76.4)
**Clinical position**	
House officer	32 (36.0)
Specialist	22 (24.7)
Senior specialist	10 (11.2)
Consultant	7 (7.9)
Senior consultant	18 (20.2)
**Specialty**	
Family medicine	9 (10.1)
Internal medicine	37 (41.6)
Paediatric medicine	12 (13.5)
Behavioural medicine	10 (11.2)
Obstetrics and gynaecology	7 (7.9)
Surgery	14 (15.7)
**Years of experience**	
1–10	44 (49.4)
>10	45 (50.6)
Mean ± SD (range)	12.5 ± 9.4 (1–30)
**Qualifications**	
MD/BMSc	35 (39.3)
Board/fellowship	54 (60.7)

SD = standard deviation; MD = doctor of medicine;

BMSc = bachelor of basic medical sciences.

**Table 2:** Distribution of responses to selected questions related to knowledge, training and experience (N = 89)

Item	Response, n (%)	
	Yes	No
1. Have you ever received any education/training for breaking bad news?	72 (80.9)	17 (19.1)
2. Do you feel that training is needed for adequate skill development in breaking bad news?	86 (96.6)	3 (3.4)
3. Are you willing to attend training regarding breaking bad news in the future?	70 (78.7)	19 (21.3)
4. Have you ever broken bad news to patients or patients’ family?	77 (86.5)	12 (13.5)
5. Did you have any bad experiences due to improperly breaking bad news?	29 (32.6)	60 (67.4)
7. Do you believe that the bad news should be delivered directly to the patients?	73 (82.0)	16 (18.0)
8. Have you ever broken bad news to patients’ family without the patient’s consent?	29 (32.6)	60 (67.4)
9. Have you ever broken bad news to patients through phone?	9 (10.1)	80 (89.9)

**Table 3:** Participants’ adherence to the SPIKES protocol (N = 89)

Item	n (%)		
	Never	Sometimes	Usually
1. **S**. Do you **set up** (plan) the interview for the patient to feel comfortable and maintain privacy?	10 (11.2)	26 (29.2)	53 (59.6)
2. **P**. Do you assess the patient's **perception** (what he already knows) about the condition?	1 (1.1)	20 (22.5)	68 (76.4)
3. **I**. Do you obtain the patient's **invitation** (ask him what they want to know)?	2 (2.2)	31 (34.8)	56 (62.9)
4. **K**. Do you give information (**knowledge**) to the patient about their condition?	2 (2.2)	11 (12.4)	76 (85.4)
5. **E**. Do you assess the patient's **emotions** with **emphatic** responses?	2 (2.2)	13 (14.6)	74 (83.1)
6. **S**. Do you explain the future **strategies**, including treatment options and prognosis?	3 (3.4)	12 (13.5)	74 (83.1)

**Table 4:** Participant’s SPIKES protocol scores (N = 89)

SPIKES score	n (%)
0	1 (1.1)
4	1 (1.1)
6	4 (4.5)
7	2 (2.2)
8	4 (4.5)
9	10 (11.2)
10	15 (16.9)
11	23 (25.8)
12	29 (32.6)

**Table 5:** Participant’s SPIKES protocol scores categories (N = 89)

SPIKES score category	n (%)
Low adherence (scores of <6)	2 (2.2)
Medium adherence (scores of 6–8)	10 (11.2)
High adherence (scores of ≥9)	77 (86.5)

**Table 6:** Association of participant’s SPIKES protocol scores categories and demographic characteristic (N = 89)

Characteristic	Adherence level, n (%)		*P* value
	Low/medium adherence (n = 12)	High adherence (n = 77)	
**Gender**			3.765
Male	3 (25.0)	42 (54.5)	
Female	9 (75.0)	35 (45.5)	
**Age**			0.662
≤40	6 (50.0)	48 (62.3)	
>40	6 (50.0)	29 (37.7)	
**Marital status**			0.015
Single	3 (25.0)	18 (23.4)	
Ever been married	9 (75.0)	59 (76.6)	
**Clinical position**			3.024
House officer	4 (33.3)	28 (36.4)	
Specialist	2 (16.7)	20 (26.0)	
Senior specialist	2 (16.7)	8 (10.4)	
Consultant	0 (0.0)	7 (9.1)	
Senior consultant	4 (33.3)	14 (18.2)	
**Specialty**			2.873
Family medicine	2 (16.7)	7 (9.1)	
Internal medicine	2 (16.7)	5 (6.5)	
Paediatric medicine	2 (16.7)	10 (13.0)	
Behavioural medicine	4 (33.3)	33 (42.9)	
Obstetrics and gynaecology	1 (8.3)	13 (16.9)	
Surgery	1 (8.3)	9 (11.7)	
**Years of experience**			1.423
1–10	4 (33.3)	40 (51.9)	
>10	8 (66.7)	37 (48.1)	
**Qualifications**			0.032
MD/BMSc	5 (41.7)	30 (39.0)	
Board/fellowship	7 (58.3)	47 (61.0)	

MD = doctor of medicine; BMSc = bachelor of basic medical sciences.
